# Editorial: GPCR in Inflammatory and Cancer Diseases

**DOI:** 10.3389/fendo.2020.588157

**Published:** 2020-09-25

**Authors:** Alain Couvineau, Rosa P. Gomariz, Yossan-Var Tan

**Affiliations:** ^1^INSERM UMR1149/Inflammation Research Center (CRI), Team “From Inflammation to Cancer in Digestive Diseases” Labeled by “la Ligue Nationale contre le Cancer”, University of Paris, DHU UNITY, Paris, France; ^2^Department of Cell Biology, Faculty of Biology and Medicine, Complutense University of Madrid, Madrid, Spain; ^3^University of Rouen Normandy, INSERM U1234 PANTHER, IRIB, Rouen University Hospital, Department of Immunology and Biotherapy, Rouen, France

**Keywords:** GPCR, inflammation, neurodegenerative diseases, cancer, neuropeptides

The G protein-coupled receptor (GPCR) family represents the largest class of membrane receptors, and displays extensive molecular diversity in terms of response to extracellular stimuli (1). The GPCRs consequently exhibit pleiotropic activities in human physiology covering cerebral, cardiovascular, digestive, pulmonary, kidney, endocrine and exocrine functions (2). Notably, they exert a pivotal role in inflammatory processes (i.e., immune cell activity, chemotaxis, angiogenesis, and tissue repair) as well as in the initiation and progression of cancer (3). Given their structural resolution and drug development, it is estimated that more than 35% of molecules prescribed in human medicine actually target GPCRs (4). Research during last decade has brought to light the importance of the GPCR family in the treatment of human pathologies associated with the most significant causes of death in the world including chronic inflammatory diseases and many forms of cancer. Very recently, the Covid-19 pandemic has revealed the importance of GPCR network related to the renin-angiotensin system (RAS). In this regard, SARS-CoV-2 infects the human cells *via* the angiotensin-converting Enzyme 2 (ACE2), an enzyme that normally inactivates angiotensin a GPCR ligand and critical cardiovascular regulator (5–7). Moreover, Relief Therapeutics is conducting a US Food and Drug Administration (FDA)-approved phase II clinical trial at New York University Langone (NYU Langone Health) to evaluate the use of Aviptadil for Covid-19-related Acute Respiratory Distress Syndrome (ARDS) (8). Aviptadil is a synthetic form of Human Vasoactive Intestinal Peptide (VIP) which exerts its functions through two GPCR receptors, VPAC1 and VPAC2 as reflected in one of the contributions below (8).

The present Research Topic assembles six review articles highlighting the importance of GPCRs in human pathological contexts. These articles represent a sample highlighting the relevance of GPCR receptors as attractive targets in the treatment of inflammatory and neurodegenerative diseases but also in cancer. These six review articles emphasize the GPCRs that respond to prototypes of several functional classes of mediators, including neuropeptides (VPAC/PACAP and orexins systems), chemokines (CXCL13 and CXCL12), hormones (melanocortin) and proteases (trypsin, thrombin…), and in the development of new innovative molecules for the treatment of the inflammatory, neurodegenerative diseases, and cancer.

## Reviews

Gomariz et al. provided an overview of Vasoactive Intestinal Peptide (VIP) and its GPCRs, VPAC1, and VPAC2 in rheumatoid arthritis (RA). VIP/VPAC axis is involved in both innate (i.e., effective anti-inflammatory action through, among other mechanisms, modifying the inflammatory profile of macrophages) and adaptive (i.e., Th2 polarization over Th1, Treg stimulation and Th17 inhibition) immunological functions. These data suggest its involvement in inflammatory/autoimmune diseases. In the context of RA, the authors highlighted the use and development of both animal models and human *ex vivo* studies in order to further evaluate the therapeutical potential of VIP. Strikingly, recent clinical studies showed a correlation between VPAC receptor expression levels as well as VIP genetic variants and the severity of the inflammatory disease. These data suggest that VIP/VPAC axis could be considered as predictive biomarkers in RA allowing a better classification of patients and a more personalized treatment strategy.

Couvineau et al. reviewed the importance of orexins in inflammatory and neurodegenerative diseases. Orexins also named hypocretins represent a new class of hypothalamic neuropeptides involved in the regulation of sleep, feeding, endocrine and cardiovascular functions which are mediated by two GPCRs termed OX1R and OX2R. The authors speculate that the orexin/receptor system could be added to the list of neuromodulators having immunoregulatory properties. The authors focused on the anti-inflammatory action of orexins in various diseases in which inflammation plays a key role, including inflammatory bowel disease, multiple sclerosis, septic shock, Alzheimer's disease, and high fat diet-induced obesity. Beside these anti-inflammatory actions, the authors reviewed the neuroprotective actions in Alzheimer's disease, narcolepsy and multiple sclerosis suggesting that orexins represent a novel therapeutic target in these multiple pathologies.

Kazanietz et al. discusses the contribution of the chemokine C-X-C motif ligand 13 (CXCL13) and its receptor, the GPCR CXCR5 in the development of cancer. They describe in detail how CXCL13/CXCR5 signaling initiates complex autocrine and paracrine cellular interactions within the tumor microenvironment. The authors detail how these activities ultimately impact cancer phenotype, including: (1) cancer cell proliferation and metastatic and dissemination functions and (2) myeloid cell and lymphocyte infiltration. This review suggests the importance of better understanding the molecular and cellular events under CXCL13/CXCR5 control, and how the knowledge could be used to evaluate the efficacy of current cytotoxic and immune-targeted therapies, as well as to identify new therapeutic targets in cancer.

García-Cuesta et al. studied the importance of the GPCR receptor CXCR4 in autoimmune diseases. CXCR4 classically known to mediate leucocyte migration and/or recruitment, has the stromal cell-derived factor-1 (SDF1), also called the chemokine C-X-C motif ligand 12 (CXCL12), as its ligand. The latter also binds another chemokine receptor named ACKR3 which can impact CXCR4 functions and is overexpressed in multiple cancer types. They describe the capability of the CXCL12/CXCR4/ACKR3 axis to regulate both immune cell trafficking and responses. This dual function provided evidence for its therapeutical value in different autoimmune and/or inflammatory diseases (i.e., psoriasis, multiple sclerosis, rheumatoid arthritis, systemic lupus erythematosus, type I diabetes, inflammatory bowel disease).

Wang et al. discusses the action of adrenocorticotropic hormone (ACTH) and α-, β-, and γ-melanocyte-stimulating hormones (MSH), collectively named melanocortins, in the regulation of immune responses. Ranging from *in vitro* to preclinical and clinical studies, this review describes melanocortin ligands and its receptors as well as their distribution and pharmacology. The authors focuse on the anti-inflammatory effects of melanocortins which are dependent or independent of glucocorticoids. The determination of signaling pathways regulated by melanocortins in immune response has improved the disease control and the life quality of patients. Moreover, the C-terminal tripeptide of α-MSH offers a novel therapeutical opportunity in the treatment of inflammatory disorders.

Sébert et al. reviewed the importance of protease-activated receptors (PARs) in two main intestinal diseases: inflammatory bowel diseases (IBD) and gastrointestinal cancers. In this unique class of receptors, ligands are tethered to the extracellular domain of the receptor but are released by the respective protease to activate the receptor. The authors describe in detail recent discoveries of the structure, and mechanisms of action of the PARs family. The PAR family encompassing PAR1, PAR2, PAR3, and PAR4, play an important role in digestive physiology. The authors describe how these pathways can be exploited as potential putative targets in IBD and gastrointestinal cancers. The development of innovative molecules targeting PARs, in particular PAR antagonist, represents a major issue in the treatment of these digestive diseases.

## Conclusions

The objective of this Research Topic was to review data which demonstrate that GPCRs, the largest membrane receptor family expressed at the cellular surface, play a significant role in healthy conditions as well as in inflammatory/autoimmune and neurodegenerative diseases, paving the way to the development of new therapeutic molecules.

## Author Contributions

All authors listed have made a substantial, direct and intellectual contribution to the work, and approved it for publication.

## Conflict of Interest

The authors declare that the research was conducted in the absence of any commercial or financial relationships that could be construed as a potential conflict of interest.
